# Recent advances in understanding gonadotropin signaling

**DOI:** 10.12703/r/10-41

**Published:** 2021-04-19

**Authors:** Livio Casarini, Manuela Simoni

**Affiliations:** 1 Unit of Endocrinology, Department of Biomedical, Metabolic and Neural Sciences, University of Modena and Reggio Emilia, Via G. Campi 287, 41125 Modena, Italy; 2 Center for Genomic Research, University of Modena and Reggio Emilia, Via G. Campi 287, 41125 Modena, Italy; 3 Unit of Endocrinology, Department of Medical Specialties, Azienda Ospedaliero-Universitaria di Modena, Via P. Giardini 1355, 41126 Modena, Italy

**Keywords:** LH, FSH, hCG, gonadotropin, FSHR, LHCGR, reproduction, endosome, signaling, allosteric modulator

## Abstract

Gonadotropins are glycoprotein sex hormones regulating development and reproduction and bind to specific G protein–coupled receptors expressed in the gonads. Their effects on multiple signaling cascades and intracellular events have recently been characterized using novel technological and scientific tools. The impact of allosteric modulators on gonadotropin signaling, the role of sugars linked to the hormone backbone, the detection of endosomal compartments supporting signaling modules, and the dissection of different effects mediated by these molecules are areas that have advanced significantly in the last decade. The classic view providing the exclusive activation of the cAMP/protein kinase A (PKA) and the steroidogenic pathway by these hormones has been expanded with the addition of novel signaling cascades as determined by high-resolution imaging techniques. These new findings provided new potential therapeutic applications. Despite these improvements, unanswered issues of gonadotropin physiology, such as the intrinsic pro-apoptotic potential to these hormones, the existence of receptors assembled as heteromers, and their expression in extragonadal tissues, remain to be studied. Elucidating these issues is a challenge for future research.

## Introduction

Gonadotropins are glycoprotein hormones regulating development and reproduction via targeting gonadal cells expressing specific G protein–coupled receptors. Gonadotropins are dimers having structural similarities and an identical common alpha subunit, and the beta subunit conserves about 70 to 80% of amino acid sequence identity in humans^1^. Receptor specificity is due to unique beta subunits encoded by follicle-stimulating hormone beta-subunit gene (*FSHB*) and luteinizing hormone beta-subunit gene (*LHB*), expressed in the pituitary. Choriogonadotropin beta-subunit genes (*CGBs*) are expressed in trophoblast cells and placenta^2,3^.

During the fertile age, the action of gonadotropins is sex-specific and its secretion is regulated by a feedback mechanism depending on levels of sex steroid hormone. In the male, FSH binds its receptor (FSHR) expressed in Sertoli cells, where the hormone exerts trophic functions fundamental for sustaining gamete production and maturation. In contrast, LH binding to its receptor (LHCGR) stimulates the synthesis of testosterone by Leydig cells^4^. In the female, FSHR expression in granulosa cells is a requisite for sustaining the monthly ovarian follicle recruitment and subsequent FSH-mediated events, such as oocyte maturation, selection, and estrogen synthesis^5^. LH modulates proliferative and anti-apoptotic signals in granulosa cells, accompanied by androgen production in theca cells, and culminates in the induction of the dominant follicle ovulation and progesterone production by luteal cells^6^. During the first trimester of pregnancy, human chorionic gonadotropin (hCG) replaces the luteotrophic role of LH in sustaining the demand for increasing levels of progesterone. Chorionic gonadotropin is encoded by a cluster of six genes, embedding two pseudogenes^7^, resulting in several slightly different isoforms and glycosylation variants acting through the LHCGR^8^. Although hCG glycoforms have different potencies in activating LHCGR-mediated signaling pathways^9^, the specific physiological functions of these variants, if any, are uncertain. It was hypothesized that they evolved in humans to optimize fetal brain development^10^.

The physiological functions of gonadotropins are exerted via multiple signaling cascades simultaneously activated in the target cells. A limited number of these pathways were known for decades, but an increasing body of knowledge emerging from recent literature is uncovering a more complex and complete view of the action of these hormones. Therefore, we provide an update on the classic view of gonadotropin physiology, reviewing recent advancements as well as unanswered questions.

## Update on gonadotropin physiology

### The “classic” pathway

One of the best-known intracellular signaling cascades activated by gonadotropins is the G_αs_ protein/adenylyl cyclase-dependent cAMP/protein kinase A (PKA) pathway. Historically, this pathway was studied using immunoassays to quantify cAMP accumulation in target cells thanks to the use of specific inhibitors and activators^11^. It was discovered that this pathway is fundamental for regulating steroidogenic events in gonadal cells^12^, as already known for the functionally similar steroidogenic cells of the adrenal gland^13^. An exception is provided by adult Sertoli cells, which cannot synthesize sex steroid hormones, and the cAMP/PKA pathway activated therein results in FSH-dependent trophic signals, sustaining cell metabolism and viability^14^. However, the activation of this intracellular signaling pathway may be triggered by all gonadotropins in target cells, where the intracellular cAMP increase is linked to other events such as trophic effects, mitotic functions, and sometimes apoptosis^14^. Downstream PKA, the cascade of kinase activations results in the extracellular-regulated kinase 1 and 2 (ERK1/2). The cAMP-responsive element-binding protein (CREB) phosphorylation is activated as well, inducing transcription of target genes, such as the steroidogenic acute regulatory protein StAR^15^. These common pathways modulating the steroidogenic signals are regulated in a cell- and developmental stage-dependent manner, and many newly discovered molecules and pathways were found to support steroid synthesis. For instance, protein kinase C (PKC) may amplify the cAMP-dependent steroidogenesis in both granulosa and Leydig tumor cells^16^, but another research documented the opposite effects of PKA and PKC on progesterone synthesis in ovarian steroidogenic cells^17^. Interestingly, a recent study found that, in granulosa cells, lisosphingolipids may induce cAMP-independent CREB phosphorylation, which, however, is not linked to the activation of steroid synthesis^18^. These data suggest that steroidogenesis is strictly under the control of gonadotropins and that the phosphorylation of a transcription factor per se is not enough to trigger the transcription of key enzyme-encoding genes. Moreover, it was recently demonstrated that the FSH-dependent activation of steroidogenic signals and folliculogenesis might be impinged by salt-induced kinases, indicating the relevance of these enzymes in regulating ovarian functions^19^. On the other hand, in luteal cells, progestational signals specifically regulated by LH via activation of the cAMP/PKA pathway involve the Wingless and Int-1 (Wnt) pathway components glycogen synthase kinase-3β and β-catenin and increase the acute progesterone synthesis in response to gonadotropin stimulation^20^. These data are representative of cell-dependent, complex signaling networks interacting with the classic cAMP/PKA pathway for supporting the synthesis of steroid hormones. New insights into the modulation of the steroidogenic pathways were provided by experiments performed using Leydig cell lines, treated with recently developed allosteric modulators of gonadotropin receptors. This experimental setting revealed that β-arrestin 2, known to be involved in G protein–coupled receptor (GPCR) internalization and sustained signal transduction^21^, may act as a negative modulator of testosterone synthesis^22^. Interestingly, steroidogenic signals can be induced by cAMP-independent mechanisms, at least in Leydig cells, where the LH-dependent phosphorylation of ERK1/2 and CREB may occur via activation of the Rous sarcoma oncogene-encoded protein (SRC)^23^ and epidermal growth factor receptor^24^.

### Differences between the FSH- and LH-mediated signals

The two gonadotropin receptors have common signaling pathways. Besides triggering the aforementioned cAMP/PKA pathway, both membrane receptors are known to trigger the activation of p38 mitogen-activated protein kinase (MAPK) pathway, an intracellular calcium ion (Ca^2+^) increase, and the recruitment of the adaptor protein “APPL1”^25–31^. The overlapping of FSHR- and LHCGR-dependent intracellular signaling pathways would be due to structural similarities between the two receptors as an inheritance persisting during the evolution of an encoding gene from an ancient common ancestor sequence^32^. Interestingly, mutations conferring constitutive activity to the FSHR may even support sex steroid–independent spermatogenesis^33,34^, suggesting that certain gonadal functions may be supported by the activation of overlapping pathways.

Although FSHR- and LHCGR-mediated signaling cascades are very similar, a number of receptor-specific pathways and target genes, which impact steroidogenesis, cell proliferation, and death, have been described. First of all, although both receptors are described as being capable of activating the phosphoinositide 3-kinase/protein kinase B (PI3K/AKT) pathway^35,36^, concerns about the reliability of data supporting FSH-induced pAKT activation were expressed^37^. FSHR-induced AKT phosphorylation would require the co-activation of insulin growth factor signaling cascades^38,39^. Moreover, FSH specifically regulates the histone H3 phosphorylation via a PKA-dependent mechanism^40^, an effect fundamental for activating the LHCGR-encoding gene transcription^41^. In turn, in granulosa cells, some FSH-dependent metabolic functions, such as the upregulation of glucose uptake and glycogen synthesis, are inversely related to LHCGR expression levels and may be impaired under LH-related ovarian pathological conditions^42^. Moreover, the production of epiregulin and amphiregulin occurs predominantly together with progesterone increase upon granulosa cell treatment with LH rather than FSH^43^. In addition, the steroidogenic effect of these hormones depends on their different capacity to activate aromatase expression^44^. These features would underlie hormone-specific physiological effects in the ovary, consisting of the androgenic and progestational actions mediated by LH and the stimulation of estradiol synthesis by FSH^45^. Finally, the two gonadotropin receptors differently modulate and impact cell death signals in ovarian cells. In a β-arrestin 1- and 2-depleted granulosa cell line, FSH administration enhanced the basal FSHR pro-apoptotic activity whereas LH treatment did not produce any effect^46^. This result was reproduced by FSHR but not LHCGR overexpression, suggesting that the control of life and death signals is due to structural features intrinsic to FSHR and LHCGR, which might underlie physiological events such as follicle recruitment, growth, and selection^46^. These functions might be the consequence of ancient duplications of genes encoding gonadotropin-like hormones and their receptors and would consist of evolutionary mechanisms optimizing the neuroendocrine control of human reproduction^47,48^. In light of these considerations, gonadotropin-specific intracellular signals and physiology rely on the structural diversity between the two receptors^49^, which achieves the maximal complexity in humans. Thus, although the FSHR and LHCGR induce partially overlapping signal transduction pathways, the two receptors mediate different and irreplaceable physiological effects.

## Recent advances

### Role of gonadotropin glycosylation

It was previously suggested that the incorporation of oligosaccharides in the structure of gonadotropins impacts intracellular signaling cascades activated by the hormones^50,51^. Gonadotropins can be naturally glycosylated to produce variants with physiological relevance as well as artificial compounds, synthesized for clinical purposes, with different glycosylations and pharmacological impact. The alpha subunit carries two carbohydrate chains joined to asparagine residues (N-linked glycosylation), and one and two are linked to the LHβ and FSHβ, respectively. Two N-linked glycosylation sites are also present in the hCGβ, which, in addition, has four glycosylated serine/threonine residues (O-glycosylation)^50,52^. This classic view has been revisited, especially in the last decade, thanks to studies deepening our understanding of the glycosylation on the function of FSH^53^, LH^54^, hCG^55^, and even of the related glycoprotein thyroid-stimulating hormone (TSH)^56^. For instance, the existence of several hCG glycoforms is well known, was reviewed in 2016, and was considered to have a pathophysiological sense^57^. It was proposed that carbohydrate chains on hCG may be used to predict pregnancy outcomes as a marker of pathologies and infertility treatment^58–62^. During the first trimester of pregnancy, the quantitative and qualitative production of hCG glycoforms is exceptionally variable, starting with hyperglycosylated forms (H-hCG) of trophoblastic origin, followed by less glycosylated isoforms^63^. Moreover, H-hCGs were found in the serum of patients affected by certain tumors^64^. Given these observations, it was supposed that H-hCG, rather than being an activator of progesterone synthesis^65^, would play a key role in inducing proliferative signals regulating trophoblast invasion and angiogenesis during the first two weeks of pregnancy as well as tumor growth^66^. This action would be exerted via cross-interaction between the hormone and the tumor growth factor beta (TGF-β) receptor^67^. However, this issue is controversial since another study found no TGF-β receptor activation by hCG and, instead, suggested the presence of growth factor contamination in the gonadotropin preparations^68^. Less glycosylated, “classic” hCG molecules have a shorter half-life than H-hCG^8^ and higher potency for LHCGR activation^9^. These features characterize hCG as a hormone optimized for inducing maternal and fetal steroidogenesis, which is fundamental for supporting massive progesterone production by the corpus luteum and the placenta, for estrogen production, and for triggering the masculinization of the male fetus^69^. hCG does also exert a TSH-like action, inducing the production of thyroid hormones acting on the TSH receptor (TSHR)^70^. Although the TSH-like actions of hCG require further research, a 2009 study suggested a link between thyroid autoimmunity and miscarriage^71^. However, conflicting data raise concerns about this hypothesis^72^. Finally, gestational hyperthyroidism has been linked to the expression of *TSHR* gene variants with hCG hypersensitivity^73^.

Although the debate over the pathophysiological impact of hCG glycoforms is far from being fully clarified, findings *in vitro* demonstrated that the glycosylation pattern of these molecules might modulate the intracellular signaling and gene expression^9^. Similarly, changes in the glycosylation and composition of glycans attached to FSH and LH, related to the stage of the menstrual cycle, were found in the serum of women of fertile age^74,75^. Thus, it cannot be excluded that glycoform-specific intracellular signals can be activated^76^. For instance, poorly glycosylated FSH molecules are more active *in vitro* than those fully glycosylated^77^ and this observation was repeatedly confirmed both *in vitro*^76,78,79^ and in mouse models^80–82^. Together with FSH glycoforms, di- and tri-glycosylated LH molecules were supposed to contribute differently to the regulation of natural ovarian cycles^54^ but these data need to be confirmed. To conclude, steps forward have been taken to elucidate molecular aspects related to gonadotropin glycobiology and the issue nourishes increasing interest of scientists and clinicians using these hormones as drugs for infertility treatment^83^. However, a clear-cut demonstration of its real impact on human physiology remains to be determined. Actually, the data available indicate that these glycoforms modulate short-term intracellular effects *in vitro*^76,79,84–86^, which are flattened in the long term^85,86^ and are extremely difficult to evaluate in humans without performing clinical trials with very large sample sizes^87^.

### Biased signaling and allosteric modulation of gonadotropin receptors

All GPCRs are activated upon hormone binding to their orthosteric site, which, in the case of gonadotropin receptors, is located in a wide horseshoe-shaped extracellular domain with leucine-rich repeats (LRRs)^88^. The binding of the ligand triggers a two-step activation mechanism, including the reshaping of the hormone conformation after contacting the LRR domain^89^. These events are followed by the interaction of the alpha and beta subunits with a sulfated tyrosine residue within the hinge region^90^. This process leads to the stabilization of ligand–receptor complexes and the inhibition of the agonistic activity of the hinge region^50^. As described above, these events are needed for triggering the G_αs_ protein coupling to the gonadotropin receptor and activation of the downstream cAMP/PKA pathway, which are preceded by conformational rearrangements of the transmembrane regions^91^. However, receptors may exist in multiple active conformations, leading to several signaling cascades simultaneously activated by coupling to other G proteins and intracellular interactors, triggering a network of simultaneous intracellular pathways^92^. Thus, particular signaling patterns may be induced by ligands displaying agonist, antagonist, or inverse agonist activities (or a combination of these) at the same GPCR, depending on the downstream endpoint evaluated, via induction of specific spatial conformations of the receptor molecule^93^.

Although these features may be due to the aforementioned hormone glycosylation variants, another mechanism that may alter GPCR signaling may be provided by single-nucleotide polymorphisms (SNPs) at the receptor level. This is the case of the common nucleotide change at position 2039 of the *FSHR* gene (2039A>G), leading to the amino acid change of asparagine to serine at position 680 at the intracellular portion of the protein (pN680S; rs6166). This SNP modulates the kinetics of cAMP activation, ERK1/2 and CREB phosphorylation, and synthesis of sex steroids^94^ and is associated with the gonadal response to FSH in males and females^95,96^.

Beta-arrestins are scaffold proteins that directly interact with the gonadotropin receptors^97^ and modulate desensitization, internalization, and recycling. They also counteract G_αs_ protein coupling to the receptor^98^ and upregulate ERK1/2 and AKT phosphorylation^99^. Moreover, the action of β-arrestins is susceptible to the phosphorylation pattern of the intracellular carboxyl-terminal end of GPCR, which differentially contributes to the recruitment of the scaffold protein, trafficking, and specific intracellular localization^100^ of ERK1/2 activation^101,102^. These data may explain why overexpression of β-arrestins is linked to attenuation of intracellular cAMP increase^103^ and may be associated with cell proliferation^46^. In particular, given the positive impact of β-arrestin functioning on tumorigenesis and cancer cell growth^104^, they have been suggested as a therapeutic target^105,106^ and promoted the development of *in vitro* systems for studying their functions^107^. Notably, β-arrestin–induced pERK1/2 activation occurs later but is more sustained than that triggered by the G_αs_ protein^108^, revealing that intracellular kinase cascades may be targeted via distinct pathways and kinetics, a molecular mechanism likely modulating specific cell metabolic events. However, β-arrestins and G proteins may also cooperate via the formation of complexes linked to the receptor, leading to temporally sustained cAMP signaling activated from internalized cellular compartments^21^. These molecular assemblies may also mediate the inhibition of G protein signaling, depending on the spatial conformation of the GPCR–β-arrestin complexes^109^. Therefore, elucidating the mechanisms biasing gonadotropin receptor signaling is of crucial relevance for developing new therapeutic approaches to certain diseases.

In the last decade, small-molecule chemical compounds able to bind and modulate FSHR-mediated signaling have been developed^110^. Increasing interest arose around allosteric ligands, which bind the receptor in a site different from the hormone-binding site^111^. According to the mode of action, these molecules are grouped into classes defined as neutral, negative (NAMs), or positive (PAMs) allosteric modulators. All of these molecules require gonadotropin binding to the receptor for exerting their action^111^ and modulate receptor-mediated signaling in the presence of the natural ligand. Interestingly, compounds acting via modulation of gonadotropin receptor assembly as homo/heteromers also have been described^112^. Among allosteric agonists, thiazolidinones are known to bind the FSHR transmembrane domain inducing the activation of G_αs_ protein-dependent pathways, similarly to FSH. However, compounds with preferential G_αi_ protein stimulatory activity also have been described^113,114^. Benzamides and dihydropyridines are known to have PAM activity at nanomolar concentrations for both the LHCGR and FSHR, activating cAMP in the presence of the bound ligand^115,116^. Tetrahydroquinolines belong to the NAM group for both gonadotropin receptors inhibiting cAMP and progesterone, but not estradiol, production and maturation of the ovarian follicle *in vivo*^22,117–119^. Finally, compounds with antagonistic activity for FSHR were found to block FSH-induced steroid synthesis^120,121^, similarly to what was recently found for the LHCGR^122^. Taken together, novel molecules with agonistic activity and able to differentially activate intracellular signaling are promising tools to be potentially used for optimizing or personalizing treatments of human infertility. Instead, molecules with NAM activity at the gonadotropin receptor, inhibiting the follicle growth without impairing steroid production, could become an option for contraceptive purposes.

New and exciting insights were provided by antibodies modulating selective activation of GPCRs via stabilization of particular hormone or hormone receptor conformations^91^. For instance, antibodies binding the FSHβ subunit were found to prevent the binding to the receptor and have protective effects against bone loss in ovariectomized mice^123^. Antibodies targeting a placental bovine hCG-like LH (equine chorionic gonadotropin), with both FSH and LH activity in other mammals, would be responsible for forming hormone–antibody complexes with increased β-arrestin–dependent signaling and activity *in vivo*^124,125^. New monoclonal and polyclonal compounds with agonistic activity for gonadotropin receptors and targeting the hinge region are currently under study^126–128^ and may represent an alternative approach to allosteric chemicals for modulating gonadal functions. Among these immunoglobulins, nanobodies against the FSHR, with small molecular weight and low predicted immunogenicity in humans, can inhibit cAMP production^129^. In conclusion, immunoglobulins are promising compounds for future therapeutic purposes, such as infertility^130,131^ and cancer^132^ treatment.

### Endosomal signaling and its physiological role

Mechanistic models explaining GPCR pathways of desensitization and recycling have greatly progressed in recent years. These functions have been known for decades^133^, and the elucidation of their physiological significance was addressed recently^134^ but remains mostly unclear. The current knowledge of gonadotropin receptor post-endocytotic processing indicates that these molecules are internalized and either recycled or directed to degradation after ligand binding and G protein activation. The activated receptor then is phosphorylated by GPCR kinases (GRKs) and recruits β-arrestins, leading to G protein uncoupling, desensitization, and internalization via the formation of clathrin-coated pits^135^. Recent studies have found that β-arrestin–mediated GPCRs internalization may also occur without uncoupling to G proteins, forming a molecular super-complex mediating sustained signaling^21,109,136^. Interestingly, most of these discoveries were provided by functional studies using *in vitro* transfected models, overexpressing the human FSHR and LHCGR, or rodent receptors, revealing that the internalization of human molecules is slower than that of murine receptors and is due to species-specific amino acids in the intracellular loop of the transmembrane region and the carboxyl-terminal tail^108,137^.

GPCR internalization is followed by the activation of a recycling pathway mediated by different endosomes or by the degradation via lysosomes^138^. Therefore, the endocytotic pathways may sort the fate of receptors impacting their density at the cell surface and the spatial and temporal regulation of intracellular signaling^139^. One of the most studied pathways involves the formation of early endosomes (EEs). These structures are characterized by the presence of the Ras-related protein 5 Rab-5 (RAB5) and consist of fusion vesicles routing from the plasma membrane and the trans-Golgi network^140^. The embedding of GPCRs in EEs is the first step of a process directing receptors to two possible fates; the first one is the degradative pathway, which is triggered by the replacement of RAB5 with RAB7, thus trafficking these units to form multi-vesicular bodies preceding protein degradation in the lysosomes. This is the pathway controlling receptor desensitization, which occurs after persistent stimulation of the GPCRs^139^. Trafficking-defective receptors due to mutations causing protein misfolding are known and may provoke reproductive dysfunctions falling within a wide range of phenotypes^138^. Interestingly, defects of genes involved precisely in EE functioning were recently associated with dysregulation of GPCR signaling and linked to pathological conditions, such as polycystic ovary syndrome. For instance, perturbation of RAB5- or the clathrin-binding protein (DENND1A)-encoding gene expression in theca cells may result in over-activation of androgen synthesis and subsequent increased susceptibility to the disease^141^. Another pathway regulating the fate of receptors involves the ubiquitin or RAB4 protein, rerouting EEs to degradation or to recycling endosomes, respectively. The latter route depends mostly on a so-called “PDZ ligand” binding sequence at the carboxyl-terminal tail of LHCGR^142^, and FSHR recycling may also occur via alternative pathways^133^. In this case, a crucial role is played by a palmitoylated cysteine at the C-tail, which is required for maintaining proper FSHR recycling, preventing excessive degradation and, likely, impairment of the cell signaling^143^.

**Figure 1.**
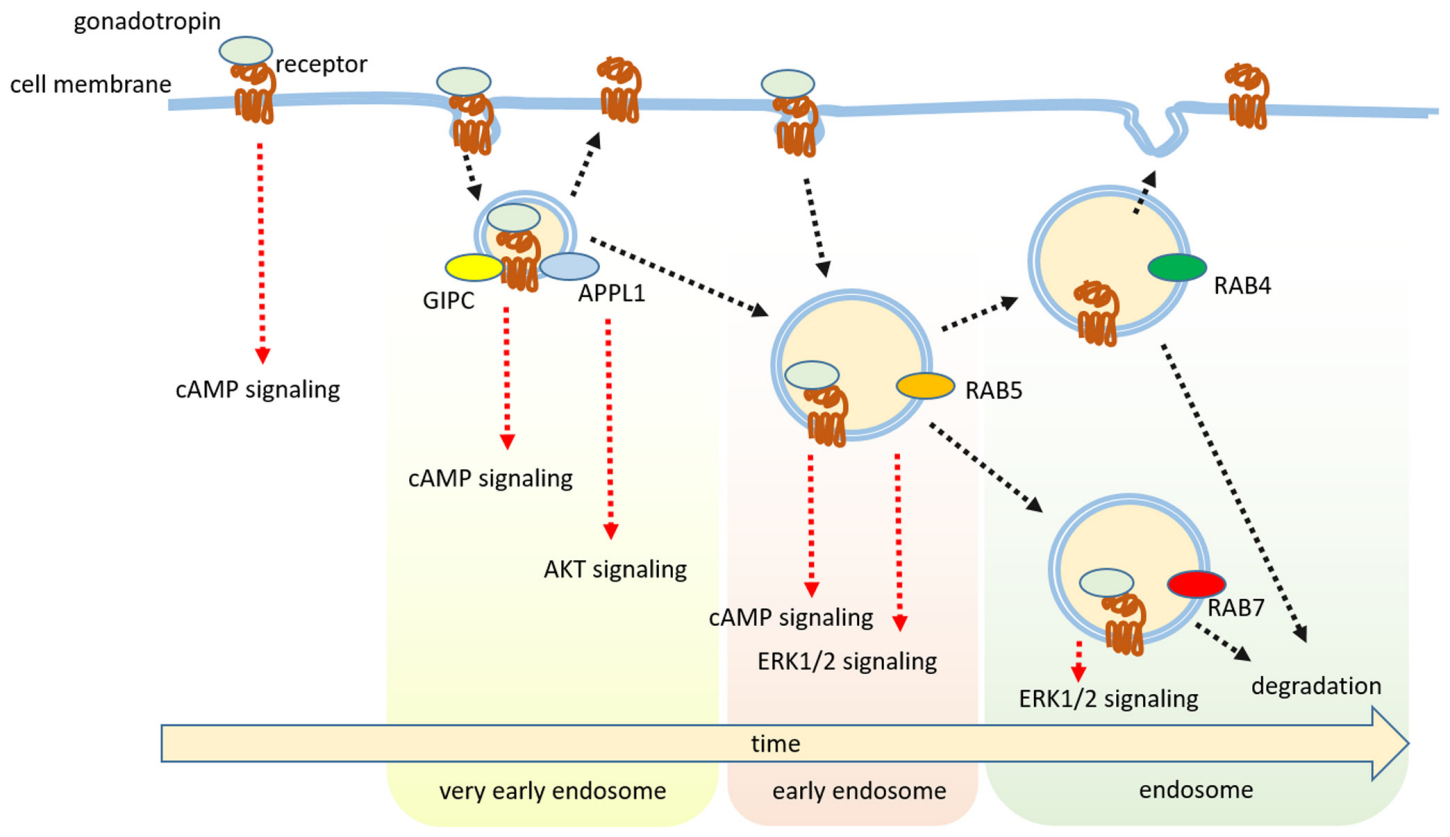
Endosomal signaling of gonadotropin receptors. Receptor bound to the hormone is internalized via different kinds of endosomal compartments, sustaining prolonged signaling, receptor degradation, or recycling on the cell surface. AKT, protein kinase B; ERK1/2, extracellular-regulated kinase 1 and 2; GIPC, “G_αi_-interacting protein C-terminus”; RAB4, Ras-related protein Rab-4; RAB5, Ras-related protein Rab-5; RAB7, Ras-related protein Rab-7.

A gonadotropin receptor recycling pathway involves a novel intracellular compartment, the very-early endosome (VEE), where LHCGR is routed through the interaction of the so-called “G_αi_-interacting protein C-terminus” (GIPC) with the PDZ protein binding domain^100^. VEE differs from the EE in that the former lacks RAB5 and is smaller, but *in vitro* removal of the PDZ domain from LHCGR re-routes the receptor to the EE^100^, inducing sustained ERK1/2 phosphorylation and revealing that these recycling pathways are interconnected. Interestingly, a similar behavior was observed for FSHR *in vitro*, using GIPC knockout without the PDZ domain, demonstrating the involvement of APPL1 in the receptor recycling through VEEs^26,144,145^. Indeed, APPL1 may interact with GIPC^146^ and sustains FSHR-mediated ERK1/2 phosphorylation, as well as AKT activation and recruitment of the 14-3-3τ adapter protein^26,144,145,147^, through a subpopulation of VEEs positive for APPL1. Interestingly, this molecule is recruited upon cAMP/PKA activation occurring via the FSHR and LHCGR itself^148^ and depletion of VEE formation results in cAMP inhibition, as demonstrated for other GPCRs^149^. However, PKA (and VEE) blockade by a specific inhibitor did not evoke similar effects, at least for LHCGR, which can induce sustained cAMP signaling, indicating a dual, receptor-specific effect of APPL1^25^. These data may be relevant for elucidating the whole functions exerted by gonadotropin receptors and their physiological impact. For instance, sustained LHCGR-mediated cAMP signaling would be required to unlock the oocyte from meiotic blockade^150^, demonstrating its effects on reproduction. In summary, gonadotropin receptor trafficking, being much more than a pathway for recycling these molecules to the cell surface, plays a crucial role in sustaining prolonged intracellular signaling (Figure 1).

### Functional differences between LH and hCG

LH and hCG are distinct molecules that have different functions^7,10,151^. Despite this knowledge, the fact that the two gonadotropins act on the same receptor and the difficulties of dissecting these differences in clinical studies^152^ support the common belief that they are two clinically equivalent hormones. Thus, commercial preparations consisting of FSH, which may be supplemented with LH or hCG or both, were developed^153^ under the assumption that hCG would provide “LH activity”^154^ during the follicular phase of the menstrual cycle in inducing oocyte maturation and ovulation^153^. In the last decade, several studies elucidating specific roles of LH and hCG were published and reviewed^3,155–157^, pinpointing the differences between the two gonadotropins.

LHβ and hCGβ have a common evolutionary origin since *CGB* genes originated in primates likely by loss of the original stop codon and repeated duplication from an ancestral *LHB* gene^158,159^. More generally, CG molecules exist first in primates^160^ but are missing in other mammals traditionally used to evaluate the action of hCG, such as mice^161–163^. Indeed, historically, LH action was typically studied using hCG in rodents. This is indicative of hCG binding capability to non-primate receptors that are structurally similar to primate LH/CG receptors^164^. Thus, it appears that in humans the complexity of the LHβ/hCGβ-encoding gene cluster is greater than that of other primates. It was suggested that this complexity might serve for supporting a more complex mechanism of placentation and highly energy-demanding brain development in humans^10^ but this remains an unproven claim. In any case, this complexity indicates a specialization occurring uniquely in the LHCGR, which, in contrast to FSHR, discriminates between two ligand-specific signals and physiological functions. This exclusive feature would rely mostly on the region encoded by the *LHCGR* gene exon 10, which may recognize and distinguish the molecular interaction between LH and hCG, modulating different hormone receptor conformations and intracellular signaling^165,166^. The role of the exon 10–encoded receptor domain was highlighted by a clinical case describing a boy with impaired development of secondary sexual characteristics due to a naturally occurring deletion of the *LHCGR* exon 10^151^. Although the phenotype at birth was clearly male, this patient presented with primary hypogonadism, which could be rescued by treatment with hCG, suggesting a possible action of the maternal choriogonadotropin inducing fetal sex determination but a lack of function of the high LH levels after birth. These data were corroborated by the discovery that the LH receptor sequence of the New World monkey *Callithrix jacchus* naturally lacks the exon 10–encoded domain. In this primate species, the pituitary produces an hCG-like molecule and not LH, thereby replacing the role of LH^167,168^ in other species in which exon 10 is present.

**Figure 2.**
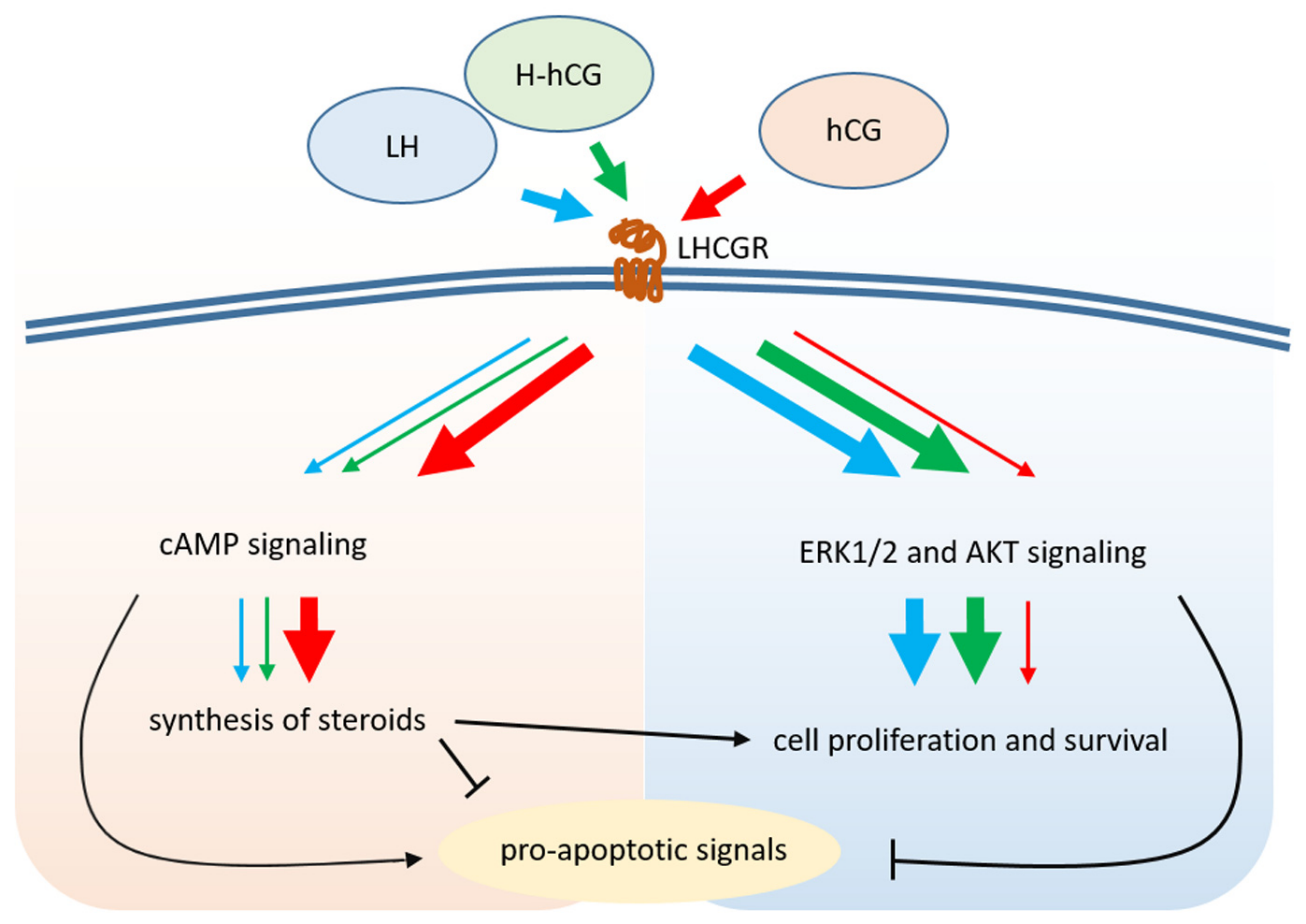
LH- and hCG-specific signals. LH and hCG induce different intracellular signaling. Whereas LH is more potent than hCG in activating proliferative and survival signals via EK1/2 and AKT phosphorylation, the choriogonadotropin acts preferentially as a progestational, inducing a more potent cAMP response. H-hCG has a relatively high proliferative potential and lower activity than hCG in activating the receptor. AKT, protein kinase B; ERK1/2, extracellular-regulated kinase 1 and 2; hCG, human chorionic gonadotropin; H-hCG, hyperglycosylated human chorionic gonadotropin; LH, luteinizing hormone; LHCGR, luteinizing hormone/chorionic gonadotropin receptor.

Evolutionary issues suggest that LH and hCG exert different roles, a concept confirmed by the nature of their physiological functions. Although both *in vitro* and clinical data indicate that these hormones could be equivalent in inducing testosterone synthesis and spermatogenesis^169,170^, reflecting the primary androgenic role of Leydig cells, different results were found in *in vitro* models of ovarian cells^171,172^. The two molecules display different intracellular signaling (Figure 2), indicating that hCG has a higher steroidogenic potential than LH and the latter induces preferentially proliferative and anti-apoptotic signals^171–174^. Moreover, they display biased agonism at the LHCGR, differentially activating cAMP production and recruitment of β-arrestin 2, likely explaining the partial agonism of LH on progesterone production^175^. Because H-hCG activates the receptor with lower activity than hCG and retains a highly proliferative potential^9^, it could be assumed that the hyperglycosylated gonadotropin modulates LH-like intracellular signaling patterns. Interestingly, the mouse receptor (Lhr) cannot discriminate qualitatively between the two human ligands^170^. This discrepancy between human and mouse receptor functioning should be due to LHCGR key residues involved in hCG/LH discrimination^165,166^, which are missing in the Lhr^176^. In any case, data from human cells support the different physiological roles of the two hormones, which would consist of potent progestational effects mediated by hCG and a fine-tuned regulation of life signals required for ovarian follicle growth and maturation for LH^3^. Moreover, clinical hormone-specific effects may be evaluated by meta-analyses of studies analyzing large datasets, oppositely to what other studies performed in a clinical^177^ or *in vitro*^84,86^ context. A proper sample size is required for dissecting the effects of the two hormones *in vivo*, which indeed turn out to be different. Although the addition of hCG to FSH treatment for assisted reproduction may optimize the number of oocytes retrieved, suggesting an effect positively modulated by steroids, LH could be beneficial for the pregnancy rate as an indirect measure of better oocyte quality^152^. These data may provide helpful insights for developing personalized protocols for assisted reproduction in specific categories of patients. For instance, poor-responder women^178^ or those requiring luteal support^179,180^ might benefit from the addition of LH and hCG, respectively, during the management of an assisted reproductive cycle^154,179^. On the other hand, gonadotropin-releasing hormone (GnRH) agonists are now the alternative option to hCG to prime ovulation and reduce the risk of ovarian hyperstimulation syndrome^181,182^, but there are no clinical data comparing the effects of LH and hCG in men.

### Pharmacogenomic use of FSH

More than two decades of studies focusing on polymorphisms of gonadotropins and their receptors revealed the existence of allelic variants linked to specific reproductive phenotypes or increased risk of developing related pathologies. In contrast to mutations^183,184^, polymorphic allelic variants are commonly widespread among the human population, achieving allele frequencies greater than 1%^185^. To date, these variants may be identified by genetic screening at relatively low costs; in some instances, they are becoming relevant for developing individualized clinical treatments.

Genetic variants of *FSHB* and *FSHR* genes are promising candidates to be characterized for pharmacogenomic approaches since they impact the ovarian response to hormone stimulation^186^. Among the roughly 20 SNPs found within the *FSHB* gene, the only one demonstrated to have physiological significance and clinical impact so far is the guanidine (G) to thymidine (T) variation 211 nucleotides upstream of the start codon (c.-221G>T, rs10835638)^47^. In men, the T homozygosity is associated with lower serum FSH levels and testicular volume than other haplotypes^187^ and decreased sperm quality^188^. However, similar findings were not always replicated by other studies evaluating spermatogenic potential and Sertoli cell number^189^. Even the opposite results were found in women^190^, suggesting possible sex-specific effects of this SNP. In females, the T allele decreases the risk of developing endometriosis, delayed age of menopause, and longer menstrual cycles than the G allele^191^. At the same time, other studies found an association between the T allele and the FSH and LH levels, idiopathic infertility, and worst clinical assisted reproduction outcome^192,193^. These data indicate a lower transcriptional activity of the T allele of the *FSHB* promoter SNP^194^, which would modulate the circulating levels of the hormone, thus impacting ovarian response.

A number of activating and inactivating *FSHR* mutations have been described within the receptor regions encoded by exons 7 and 10^184^. Loss-of-function *FSHR* mutations may lead to pathological conditions, such as amenorrhea, in women^186^ and may be linked to infertile or subfertile phenotypes in men^131,195,196^. *FSHR* mutations overall are rare, and several *FSHR* SNPs may have been characterized and analyzed for their associations with the gonadal response. Among the best-known common FSHR variants, the rs1394205 (c.-29G>A) in the *FSHR* promoter, as well as the two exon 10 SNPs rs6165 (c.919G>A) and rs6166 (c.2039G>A) usually in linkage disequilibrium, have been associated with the gonadal response to FSH. In particular, the rs6166 variant is linked to the asparagine-to-serine amino acid change at position 680 of the protein chain (p.N680S) and is located in the C-terminal region of the receptor. This substitution adds a potential phosphorylation site, increases early intracellular signaling, and impacts the kinetics of steroid synthesis^94^. In practice, homozygous individuals carrying the amino acid serine are less “sensitive” to FSH and show higher serum FSH levels and longer menstrual cycles than pN680S N homozygous women^197^. A role for FSHR SNP in male fertility has also been proposed^198^. Cases of idiopathic infertility related to this SNP were described in men^199^. However, these findings were obtained after analysis of the cumulative effects of the FSHR p.N680S SNP and other SNPs, revealing the overall mild but detectable impact of the polymorphism on male reproduction^196,199^. In men, the impact of the SNP rs6166 in modulating the action of FSH was eventually characterized by a pharmacogenetic study where sperm DNA fragmentation of idiopathic infertile patients improved after prolonged treatment with the gonadotropin^96^. Therefore, the p.N680S phenotype may be a marker of sperm quality.

Studies analyzing the allele frequencies of *FSHR* exon 10 SNPs should take into account the expression level of the receptor protein given that the polymorphism c.-29G>A modulates the transcriptional activity of the gene^200^ and is linked to genotype-specific gonadal functions in both males^201,202^ and females^203–205^. In particular, associations between reproductive parameters and the c.-29G>A SNP were found when analyzed together with the p.N680S FSHR polymorphism^201^, revealing the cumulative effects of these hot spots. However, the opposite results, which likely indicate a weak clinical impact of the SNP per se, have also been reported^206–208^. Further studies using larger datasets are required to highlight the impact of this genetic variant on fertility. Therefore, the combined evaluations of several *FSHR* and *FSHB* SNPs are determinant to indicate how the whole haplotype is linked to specific endocrine phenotypes.

Although each FSHR SNP was linked to particular endocrine phenotypes in some studies, the combined analysis of several alleles revealed the real impact of specific haplotypes in increasing the risk of infertility^197,199^. These analyses should account for limitations intrinsic to each study. Different results due to ethnicity-related^201,209^ or sample size^210^ issues may occur, leading to the requirement of independent confirmation of the findings. Other limitations may be linked to the study design since weak selection criteria and different or unspecified primary endpoints provided non-comparable results^199^. Extensive interventional studies are needed to more deeply explore the therapeutic benefit of a pharmacogenomic approach to gonadotropin treatment. Nevertheless, after about two decades of small studies on markers of the gonadal response, scientific support for the development of a pharmacogenetic approach to personalized hormonal treatments is now available. Given the dramatic cost reduction for genetic testing in recent years^211^, conditions are now favorable for SNP analysis before clinical treatments of infertility.

## Unanswered questions

### Gonadotropin-driven cell death

Although steps forward have been made in the elucidation of gonadotropin physiology over the last three decades, several unknown aspects of their function remain. One of the most underrated issues related to gonadotropin signaling is the activation of pro-apoptotic signals^14^. Gonadotropins are classically linked to proliferative and survival signals, reflecting their roles in supporting gametogenesis. On the other hand, these molecules upregulate the phosphorylation of mitogenic/anti-apoptotic signals induced via ERK1/2 and AKT and through activation of several different intracellular pathways, including the positive impact on steroid hormone production, supportive of reproduction. In fact, convincing data confirmed that FSH and LH mediate signals required for sperm and somatic cell life in the male gonad^212^. Interestingly, the textbook knowledge of ovarian physiology is that follicular dominance is a process regulated by pro-apoptotic stimuli occurring after the decrease in serum FSH levels. This event would be responsible for declining proliferative signals occurring in individual follicles, which become atretic. The proliferative effect of gonadotropins is widely described in the scientific literature, where a number of studies describe how FSHR and LHCGR may activate life signaling pathways and, in certain cases, have even a tumorigenic potential^5^. However, pro-apoptotic stimuli delivered by gonadotropins via cAMP-related pathways were also described^213–215^. These effects would be triggered by relatively high intracellular levels of the second messenger, likely resulting in p38 MAPK and p53 activation^216,217^ together with steroidogenic signals^218^ and inducing cell death. From this point of view, the pro-apoptotic potential of gonadotropins would rely on the capacity to increase cAMP, a property previously demonstrated for FSH and hCG in certain experimental conditions *in vitro*^46,172^. In particular, persistently high FSHR expression levels and the lack of steroid hormones could be linked to insufficient inhibition of death signals^172^, a possible explanation for the lack of consistent human ovarian steroidogenic cell lines expressing this receptor^46^. This condition is hardly obtainable *in vivo* in the ovary, where the FSH-induced signals are translated to estrogen production and FSHR is transitorily expressed in the follicles. On the other hand, steroids activate anti-apoptotic signals^172,219^, as obligatory regulators of folliculogenesis^220^, even inducing multi-follicular maturation during assisted reproduction^221,222^, where relatively high gonadotropin doses are administered. However, death signals delivered through FSH may play an essential role in the ovary. They could be involved in the selection of the dominant follicle, inducing steroidogenesis and atresia of those follicles expressing relatively high levels of FSHR^215^. The activation of cAMP is due to the preferential coupling to the G_s_ protein when FSHR is maximally expressed^215,223^ in granulosa cells at the early/mid-antral stage of the menstrual cycle. Interestingly, oocyte maturation might depend on the capability of the membrane G protein–coupled estrogen receptor (GPER) to reprogram cAMP/death signals, linked to FSHR, into proliferative stimuli. The anti-apoptotic role of GPER in the ovary is exerted through the physical interaction with the FSHR, leading to the formation of heteromers activating the AKT pathway upon FSH binding^215^. These data indicate that the temporary succession of pro- and anti-apoptotic signals is orchestrated, at least in part, by interacting gonadotropin and steroid hormone membrane receptors, playing an essential role in female reproduction. Further studies are required to understand how these molecules act in concert to regulate folliculogenesis. Membrane receptor heteromers might be markers of ovarian cell proliferation^104,215^ to be targeted by drugs for infertility and cancer treatment.

### Receptor heteromers *in vivo*

Several GPCRs are known to form homo- and hetero-meric functional units consisting of the molecular association between the same or different but structurally similar receptors^224^. It is reasonable that GPCRs interact with molecules having an overall similar structure, as previously demonstrated for receptors of other pituitary^225^ and non-pituitary^226^ hormones. In particular, FSHR–LHCGR heteromers were demonstrated *in vitro*^227,228^, where they are classically overexpressed in transfected cell lines and analyzed using imaging methods^229,230^. Recently, the GPER has been indicated as a novel interacting partner of both FSHR and LHCGR in ovarian cells^215^. The physiological implications of these heteromers are not fully understood. It was proposed that they mediate GPCR functional diversity, highly relevant for human physiology^231^. In particular, they should be involved in the development of competent oocytes during the normal menstrual cycle, reprogramming gonadotropin receptor–mediated death signals in proliferative stimuli^215,232^. Impairment of hormone-dependent signaling due to FSHR–LHCGR heteromer formation was reported in the transiently transfected human embryonic kidney (HEK293) cell line^233^, further providing the rationale for the assembly of heteromers. However, the existence of naturally formed gonadotropin receptor heteromers, as a general feature of GPCRs, is not widely accepted^226^. In fact, this concept may be weakened by the difficulties in demonstrating heteromers in primary cell cultures, unspecific signals, or elevated background noise due to biosensor-tagged receptor overexpression obtained in an artificial system *in vitro* as well as the doubt that agonist-induced signals can be generated by heteromers or conformational changes within preassembled groups of receptors^234^. Therefore, the fact that gonadotropin receptor heteromers may occur *in vivo* is under debate. A step forward was taken by obtaining data in genetically modified mice coexpressing binding-deficient and signaling-deficient LH receptors^235^. These experiments were based on a concept previously developed and demonstrated *in vitro*; binding-deficient receptors triggered cAMP activation through the interaction with a receptor, defective in signal generation, bound to hCG^236^. Even in mice, LH was able to trigger the activation of the cAMP/PKA intracellular signaling cascade via intermolecular cooperation between the two receptors^235^. This is a mechanistic proof of gonadotropin receptor interaction *in vivo*, which presumably occurs even in the human granulosa cells. In the ovary, LH-like signals could be induced via FSH binding to FSHR during the window of receptor coexpression^232^. However, results concerning the existence of FSHR–LHCGR transactivation were also provided, although stably transfected HEK293 cells used in this study were not a physiological model^237^. This issue merits further studies, and alternative experimental approaches may be essential in demonstrating gonadotropin receptor heteromers and complementarity in a physiological context. For instance, bivalent ligands able to bind receptor dimers or super-resolution imaging analysis could be extremely helpful^238^.

### Extragonadal gonadotropin receptors

By definition, gonadotropins act in the gonads by binding to receptors specifically expressed therein as a requisite for maintaining endocrine signal specificity and proper metabolic functions. This classic view was recently challenged by several studies describing the expression of functional ectopic gonadotropin receptors, mainly FSHR, supposed to play a significant role in non-reproductive processes. These effects include bone loss, obesity, and cardiovascular and cancer risk, which could be triggered by extragonadal FSHR activated by high FSH levels^239^, such as those occurring after menopause. Lhr expression was found in the mouse adrenal cortex^240^, reflecting previous data demonstrating the presence of LHCGR expression in adrenal glands of postmenopausal women^241^. Aberrant LHCGR expression in the adrenal gland would be linked to cortisol synthesis and Cushing’s syndrome^242,243^. Functional LH receptors were also found in uterine tissues of human^244^ and farm animal models under physiological conditions^245,246^. Surprisingly, immunoreactivity to LHCGR was found even in human brain microglial cells and was positively correlated to an increased risk of developing Alzheimer’s disease^247^. More recently, the presence of LHCGR was suggested in the retina, where LH and hCG would have vascular endothelial growth factor–like functions^248^.

Functional FSHRs were found in endometriotic lesions, where FSH may induce *CYP19A1* expression and estrogen production^249^. This is not the only study describing ectopic expression of this receptor; in this regard, an increasing amount of data from human and animal models has been published in the last two decades. Genetically modified or antibody-treated mouse models elegantly suggested that FSH-induced signals modulate bone mass and adipose tissue^123,250^. Around these issues, several studies described a link between FSH, osteoporosis, and increased fat mass that occurs after menopause^251–258^. Similar conclusions were achieved by *in vitro* studies using human cells^259,260^, although the existence of direct causality between gonadotropin receptor and the physiological effect was questioned^261^. Relatively low levels of FSH were associated with increased cardiometabolic risk and cardiovascular diseases in the Chinese population^262^, and the rationale of this issue is provided by the angiogenetic support exerted by FSH via the PI3K/AKT pathway^263^. Finally, it was demonstrated that FSHR is expressed in tumor vessel cells upregulated by FSH^264–266^. These accumulating experimental data seem to clearly debunk the classic view of gonadotropin physiology, potentially leading to new diagnostic, therapeutic, and prognostic opportunities. For instance, the use of anti-FSH antibodies for bone- and fat-specific targeting was proposed for clinical testing^267^. However, the issue is highly debated, and several objections, including the low reliability of the antibodies used for investigating FSHRs in non-canonical tissues^268^, firmly oppose the existence of extragonadal FSHRs and LHCGRs^269^. These data would rely on the discrepancy between mRNA and protein level detected in the analyzed samples, suggesting that they could be an artifact due to suboptimal antibody binding^270^ and challenging Western blotting^271^. Missing control experiments of target mRNAs by DNA sequencing^263^ put further concerns on the reliability of the detection. Together with the aforementioned experimental issues, the opposite results were also found. For instance, a decrease in body fat was not found in mice and humans maintained under gonadotropin production blockade by GnRH agonist/antagonist^272–274^. Finally, studies revisiting previous experimental conditions used for detecting extragonadal FSHRs in the human umbilical vein endothelial cells failed in replicating results^275^, and several technical concerns arose when ectopic gonadotropin receptors were evaluated in other tissues^269^. In conclusion, the concept of extragonadal FSHRs remains highly controversial. Genetically modified mice may not be the best tools for assessing this issue. Interventional studies in humans will be the only way to demonstrate direct causality between gonadotropin receptors and the abovementioned effects.

## Conclusions

Significant steps forward in the elucidation of gonadotropin signaling and regulation have been made in recent years. The current knowledge addressed most of the questions of the past, revealing novel aspects of FSH, LH, and hCG physiology and providing the potential for new therapeutic applications. Despite these advances, many unanswered questions remain, and the full potential of gonadotropin action, the presence of FSHR–LHCGR heteromers *in vivo*, and the role of extragonadal receptors warrant further research. Studying these aspects is vital for further scientific and clinical progress that could benefit human reproductive health.
